# Supplementary figures and images for: Single-nucleus RNA-sequencing in pre-cellularization Drosophila melanogaster embryos
Source: PLoS One. 2022 Jun 24;17(6):e0270471. doi: 10.1371/journal.pone.0270471 (PMC9232161; doi:10.1371/journal.pone.0270471)

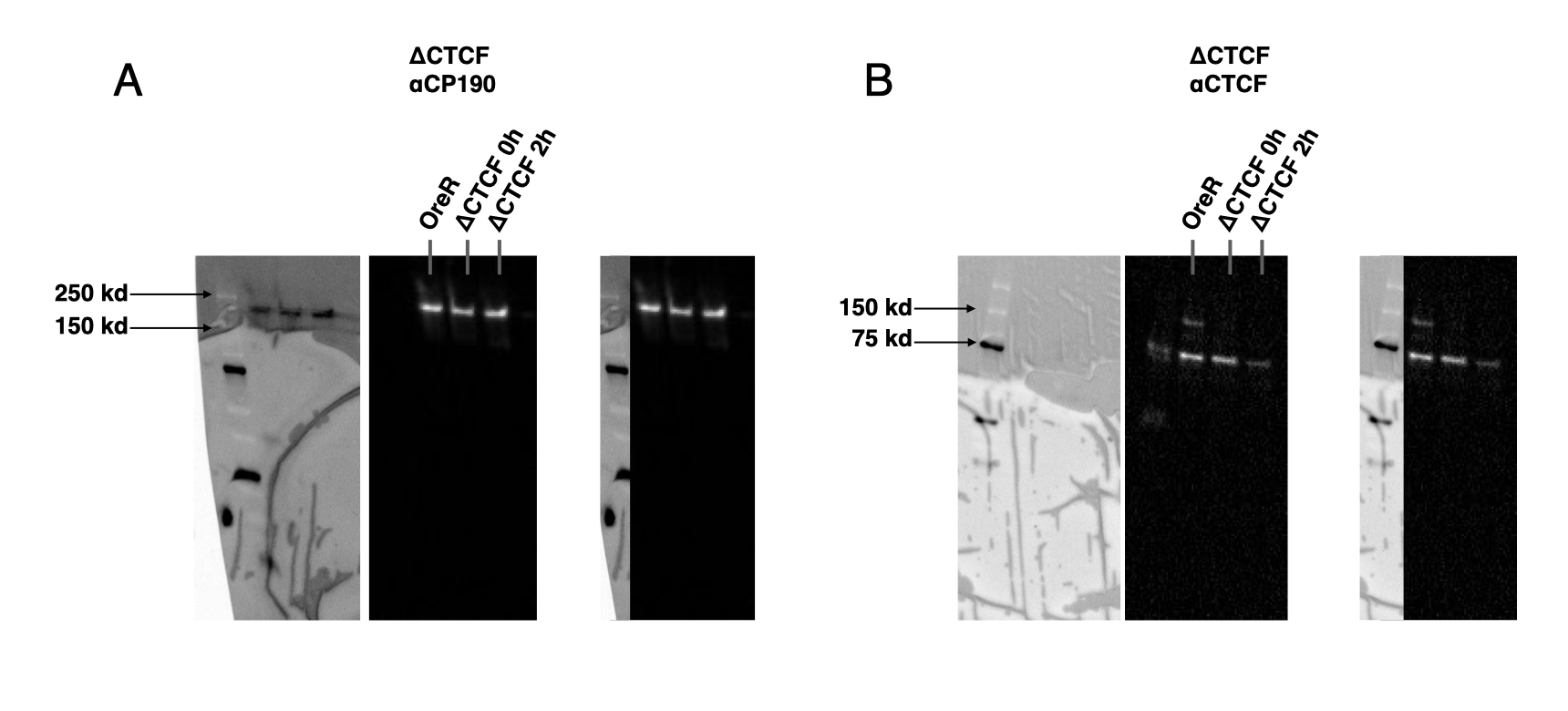

Supplement: S1 Fig — (A) Western blotting of OreR, 0h and 2h dCTCFmat-/- embryos using an antibody to Cp190, another insulator protein, as a control. (B) Western blotting of OreR, 0h and 2h dCTCFmat-/- embryos using an antibody to dCTCF. The 2h embryos were aged for an additional 2 hours with the majority of the embryos representing nuclear cycle 14, the same time point at which we conducted single-nucleus RNA-sequencing. A cross-reactive band appears at approximately 75 kd, with the dCTCF band appearing at approximately 130 kd. (TIF) [file pone.0270471.s002.tif]

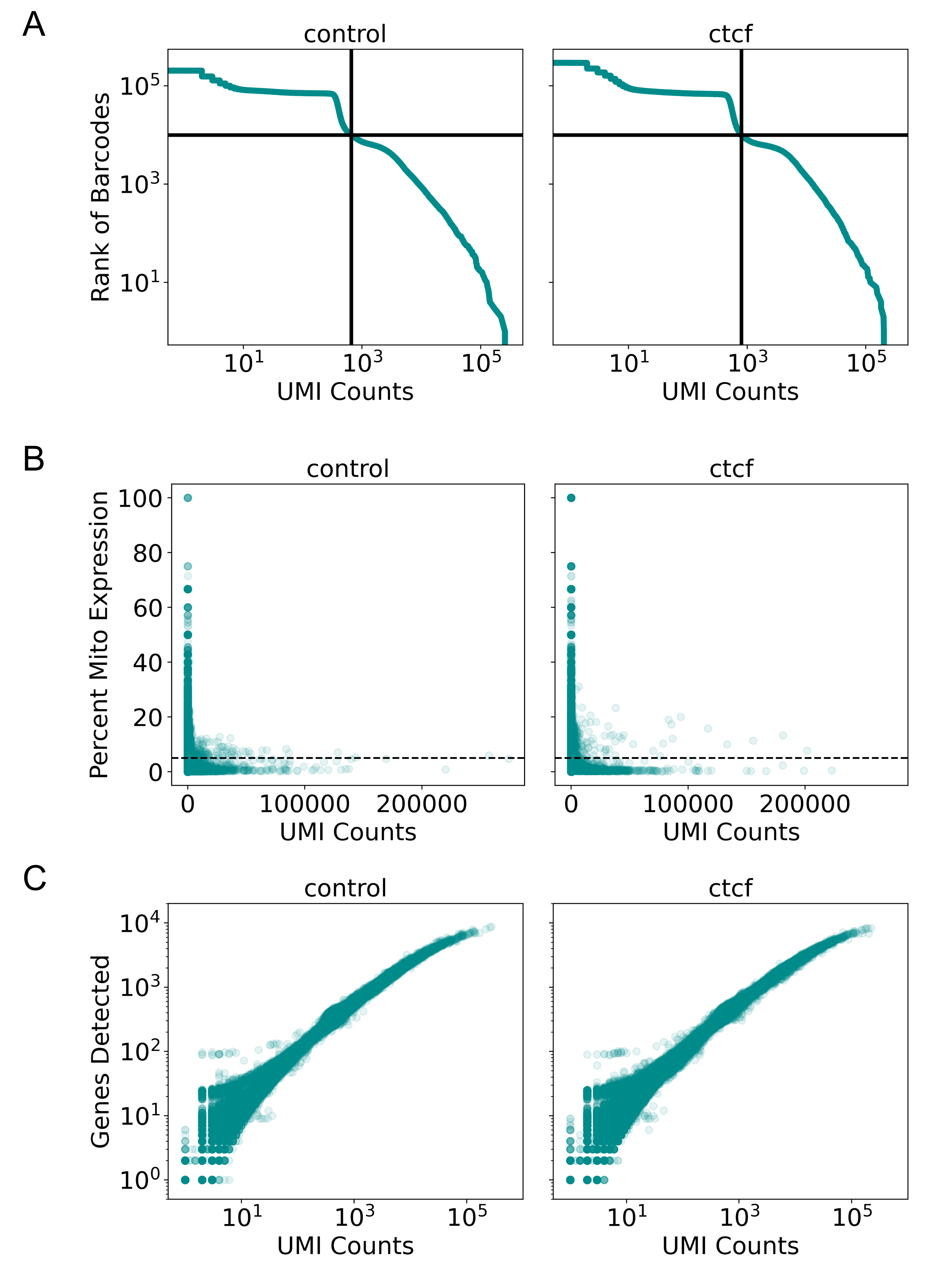

Supplement: S2 Fig — (A) Knee plot for barcodes ranked by the number of UMIs versus UMI counts for control (left) and dCTCFmat-/- (right) experiments. Black line indicates the position of the 10,000th (expected number of cells) on each axis. (B) Percent mitochondrial expression per nucleus in control (left) and dCTCFmat-/-(right) nuclei. Dashed line represents 5% mitochondrial expression, or the cutoff used for filtering the data. (C) Number of genes detected per nucleus by UMI counts in control (left) and dCTCFmat-/-(right) nuclei. (TIF) [file pone.0270471.s003.tif]

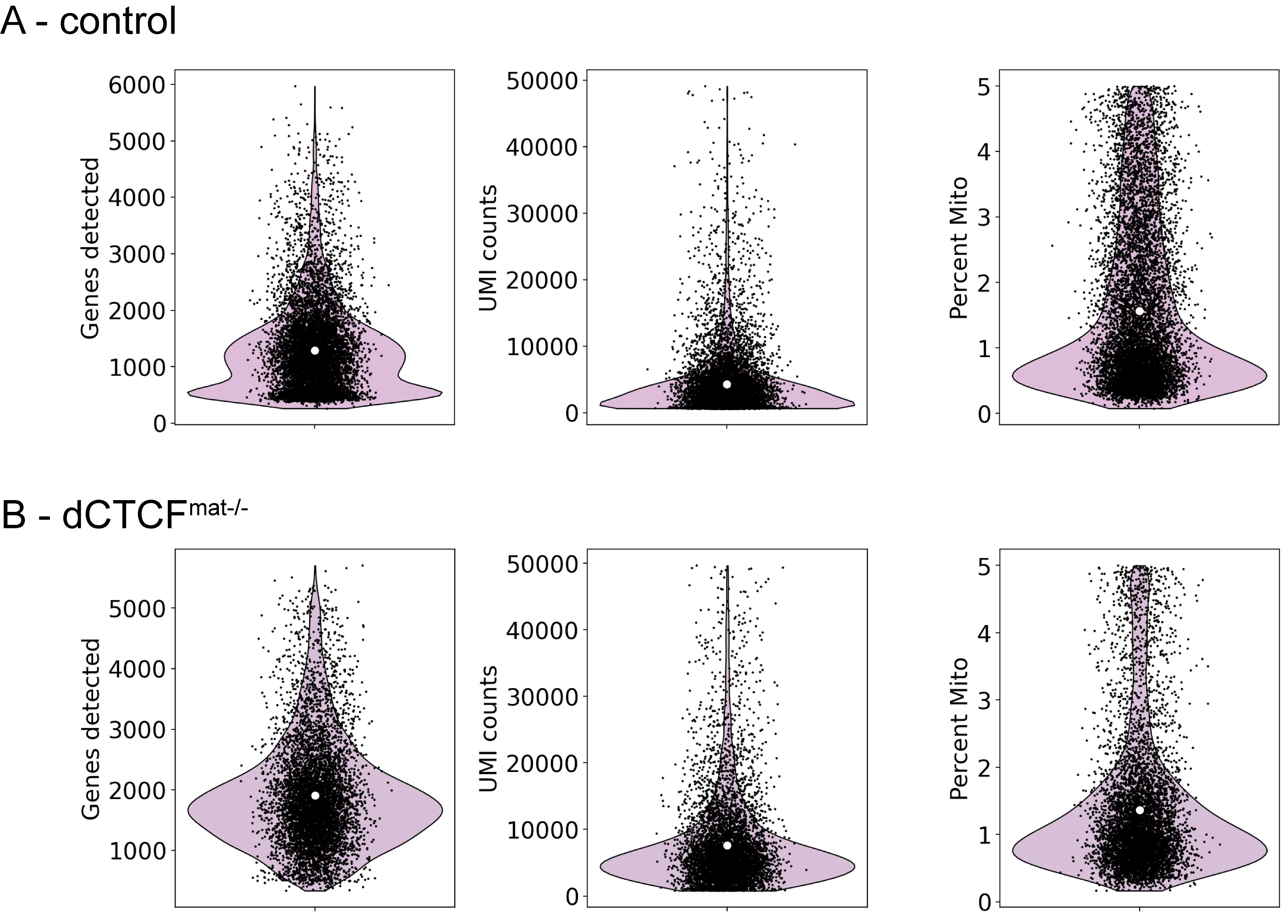

Supplement: S3 Fig — Number of genes detected (left), UMI counts (middle), percent mitochondrial expression (right) per nucleus after filtering in (A) control and (B) dCTCFmat-/- experiments. (TIF) [file pone.0270471.s004.tif]

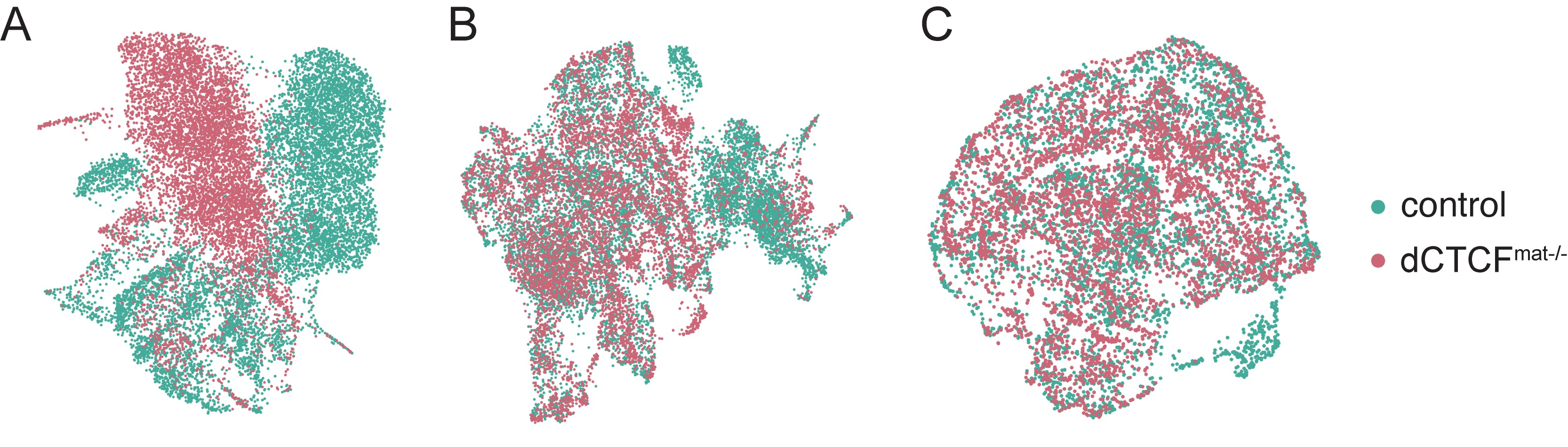

Supplement: S4 Fig — Two-dimensional UMAP embedding of control (teal) and dCTCFmat-/- (pink) nuclei (A) before, (B) after batch correction using scVI, and (C) after removing low quality nuclei. (TIF) [file pone.0270471.s005.tif]

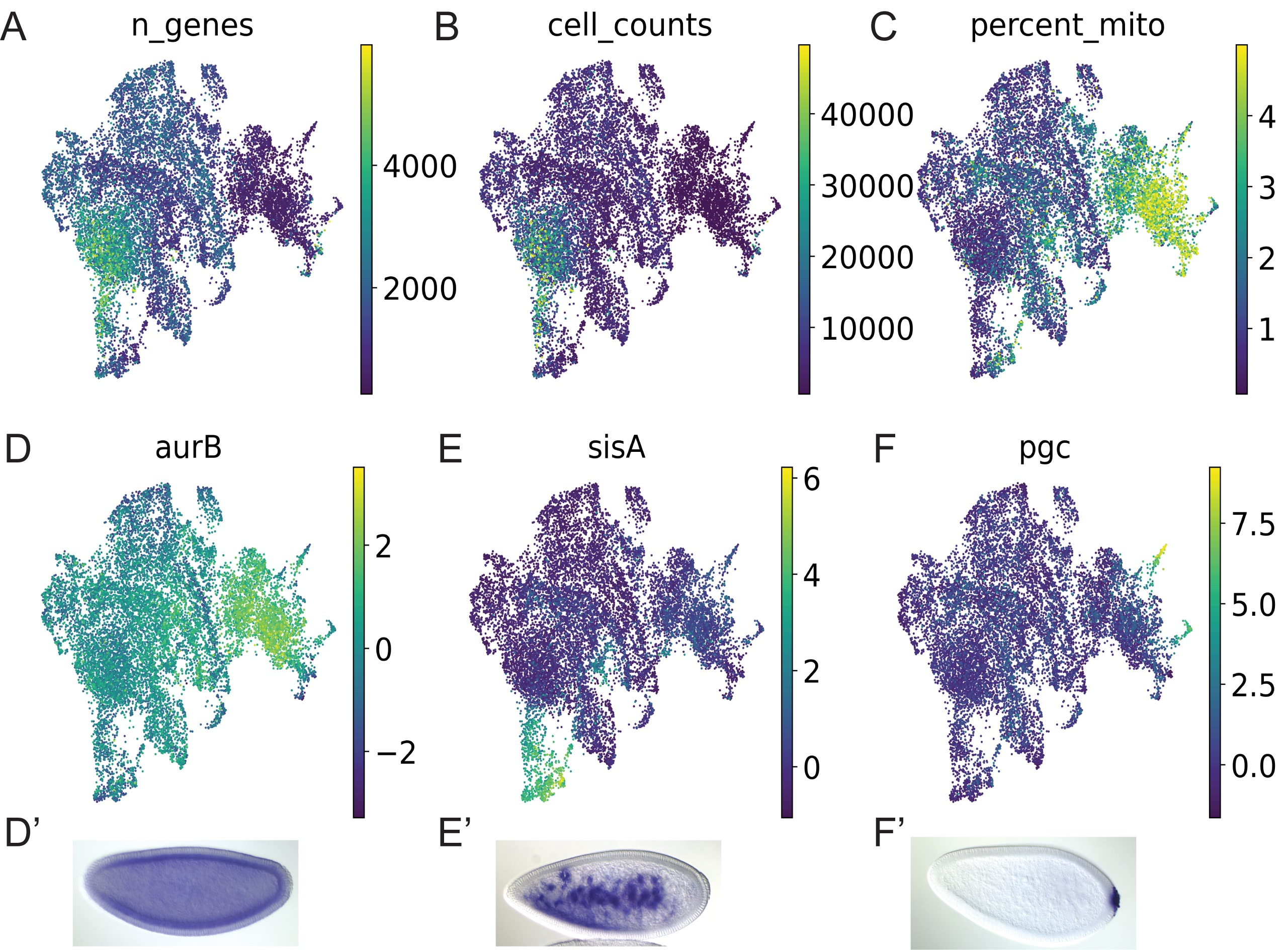

Supplement: S5 Fig — Two-dimensional UMAP embedding of nuclei before additional filtering colored by (A) number of genes detected, (B) UMI counts, (B) percent mitochondrial expression. (D-F) log(scvi normalized expression) of three genes with representative in situ hybridizations below for (D) cell cycle gene aurB, (E) yolk nucleus marker, sisA (F) and pole cell marker pgc. (TIF) [file pone.0270471.s006.tif]

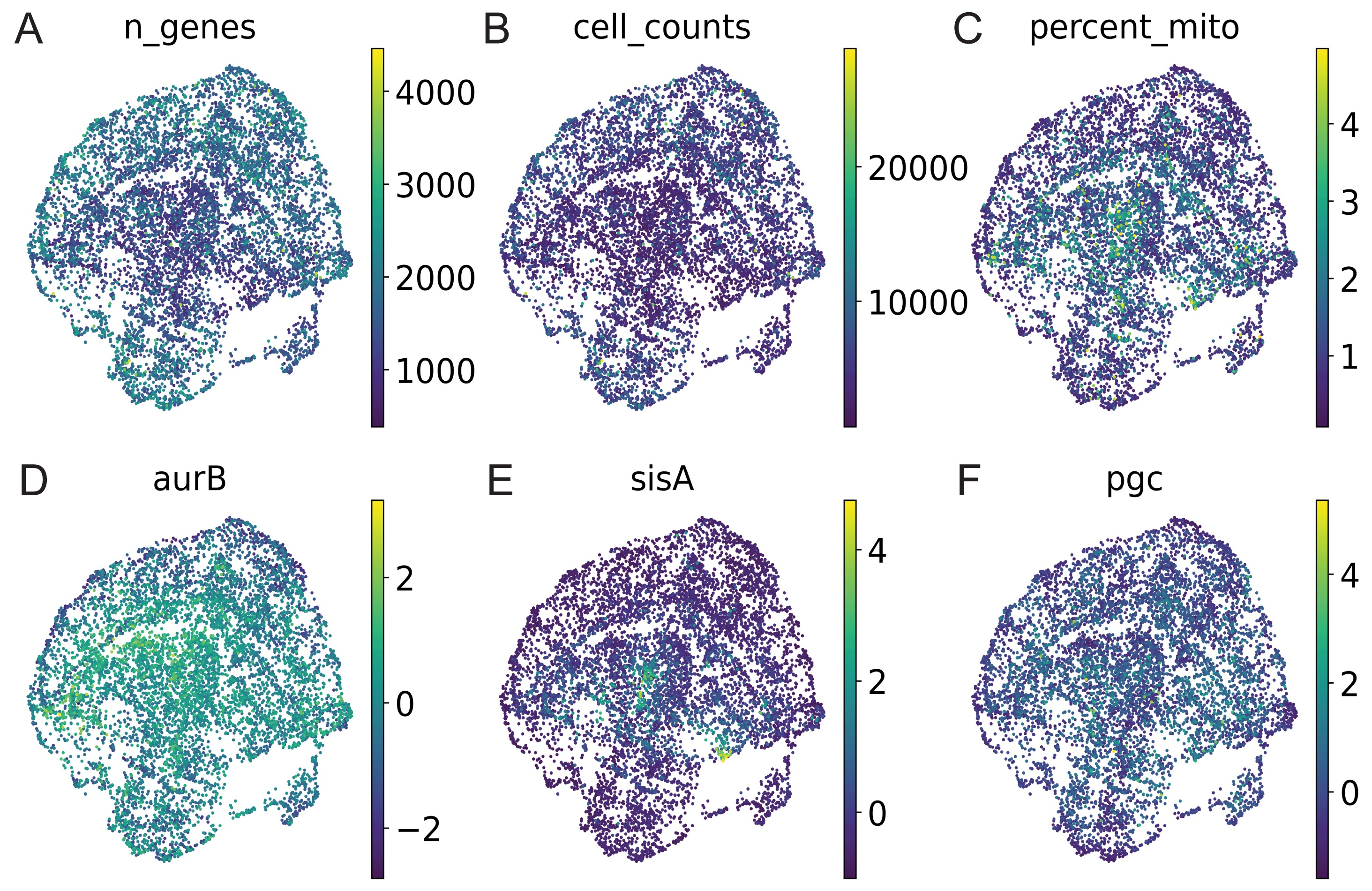

Supplement: S6 Fig — Two-dimensional UMAP embedding of nuclei after removal of clusters with high percent mitochondrial expression, aurB expression, sisA expression, and pgc expression colored by (A) number of genes detected, (B) UMI counts, (C) percent mitochondrial expression. (D-F) log(scvi normalized expression) of three genes with representative in situ hybridizations below for (D) cell cycle gene aurB, (E) yolk nucleus marker, sisA (F) and pole cell marker pgc. (TIF) [file pone.0270471.s007.tif]

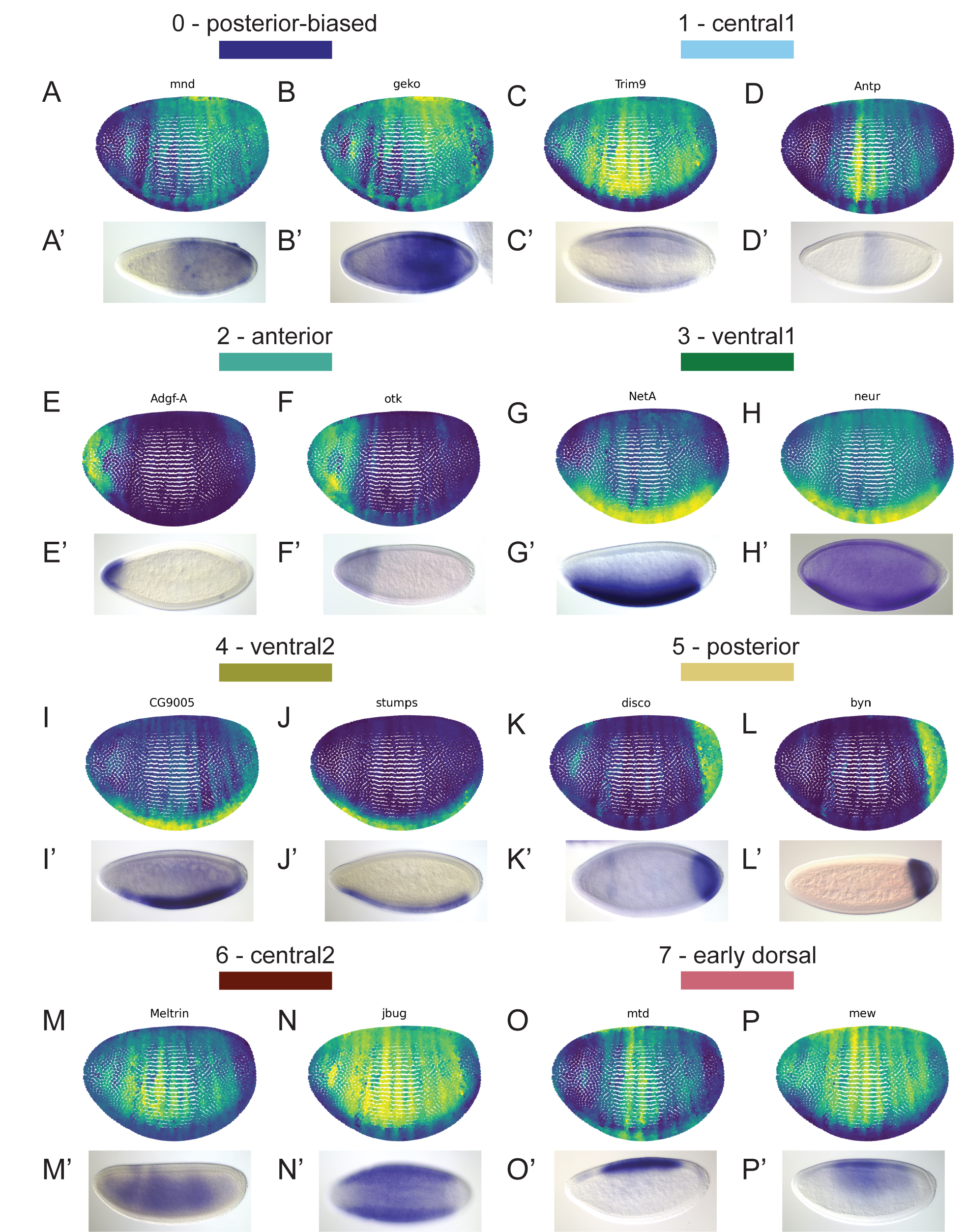

Supplement: S7 Fig — (A-P) Representative virtual (top, A-P) and Berkeley Drosophila Genome Project (bottom, A’-P’) in situ hybridizations for additional marker gene expression within each cluster as indicated. This supplemental figure accompanies Fig 2 in the main text. (TIF) [file pone.0270471.s008.tif]

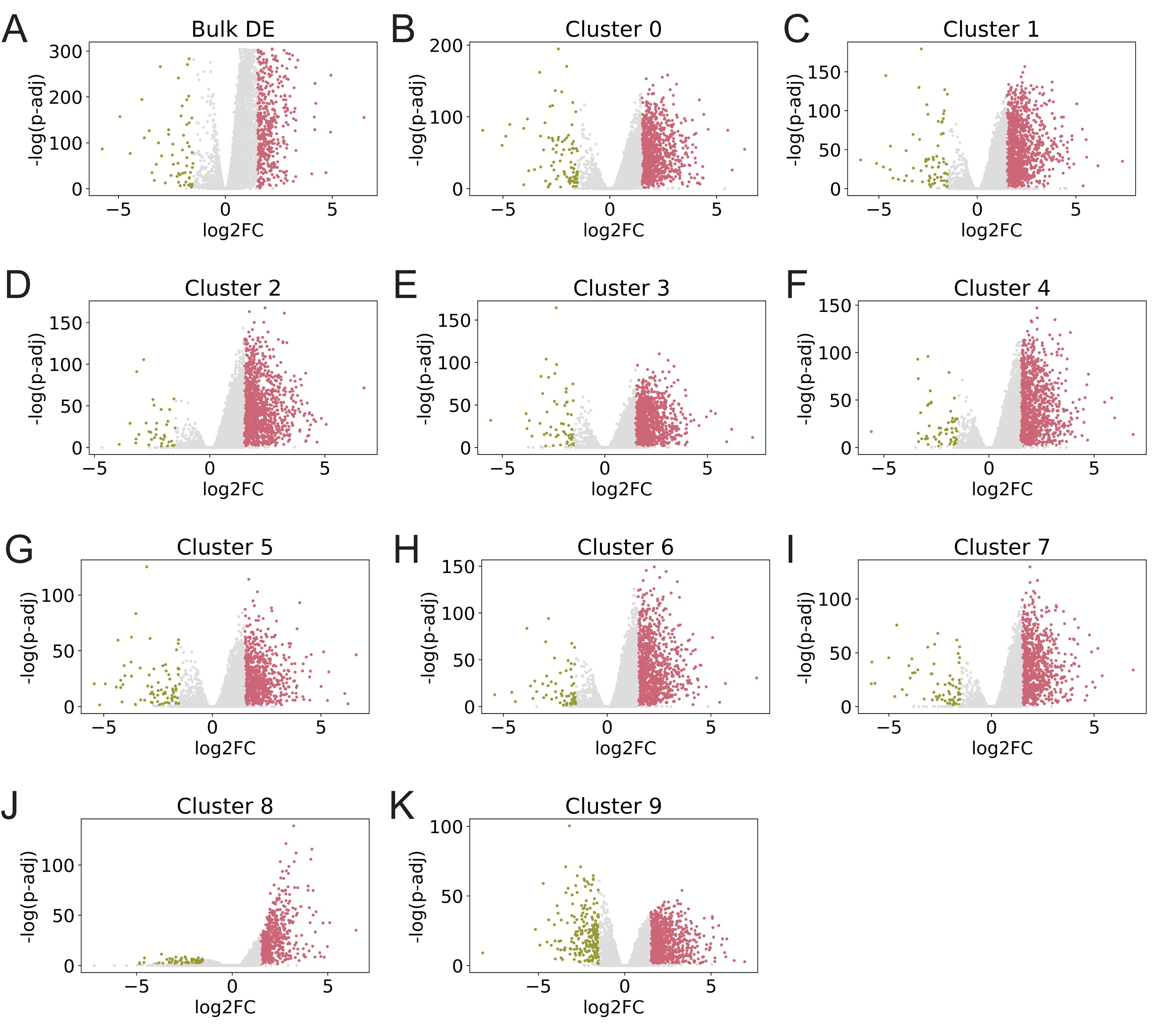

Supplement: S8 Fig — Volcano plots of log2FC (log2(fold-change)) by the log of the adjusted p-value (p-adj) for differential expression calculated in bulk (top middle) and in individual clusters as indicated. Colored dots indicate genes with significant differential expression, an absolute value of log2FC > = 1.5 and p-adj < 0.05. Significantly down-regulated genes are indicated in green, significantly up-regulated genes in pink, and non-significantly differentially expressed genes in light gray. (TIF) [file pone.0270471.s009.tif]

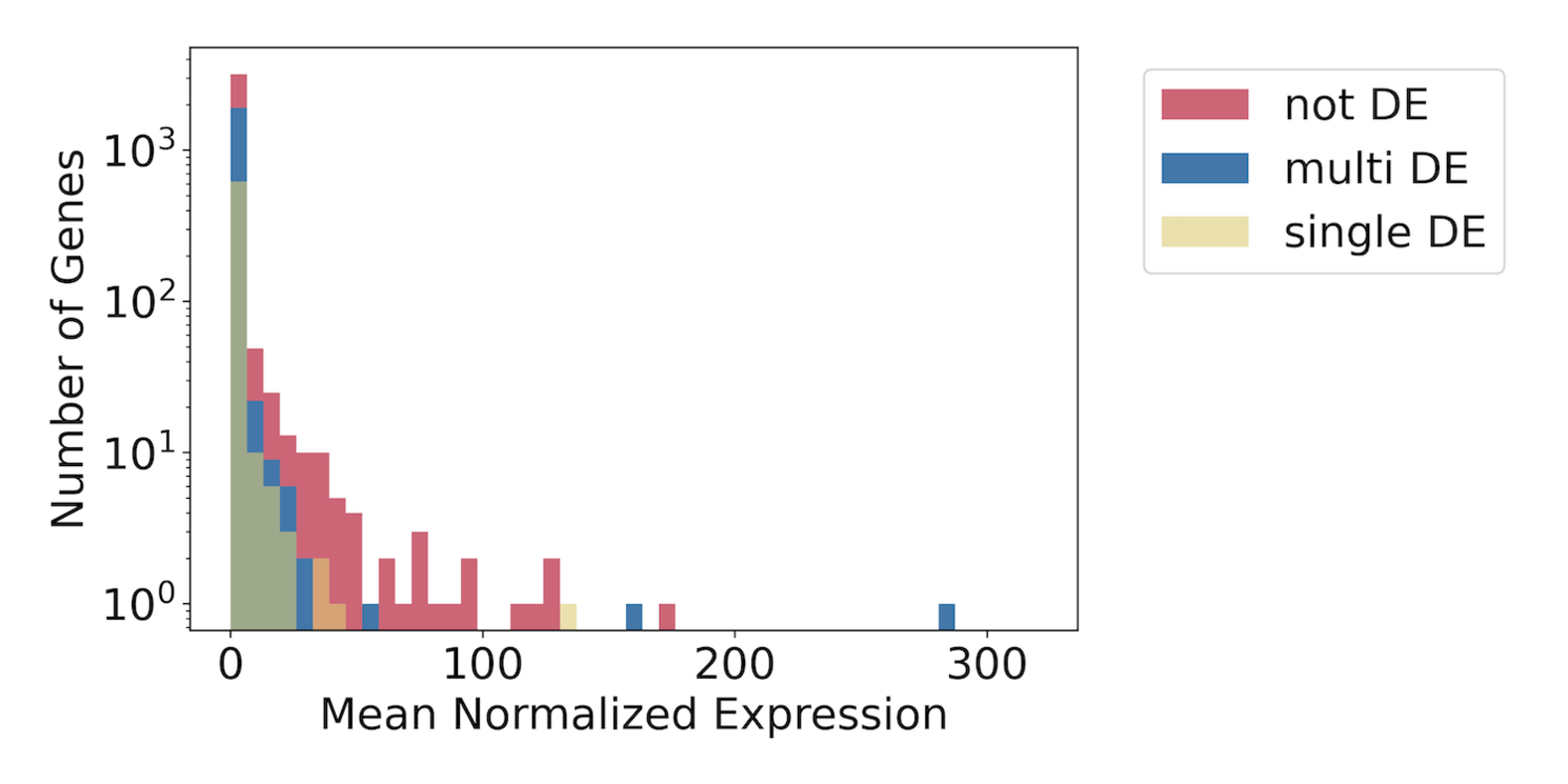

Supplement: S9 Fig — Histogram of average gene expression in transcripts per million (TPM) of differentially expressed genes in one cluster (yellow), differentially expressed in multiple clusters and/or in bulk (blue), and non-differentially expressed genes (red). Each count on the y-axis represents a single gene. (TIF) [file pone.0270471.s010.tif]

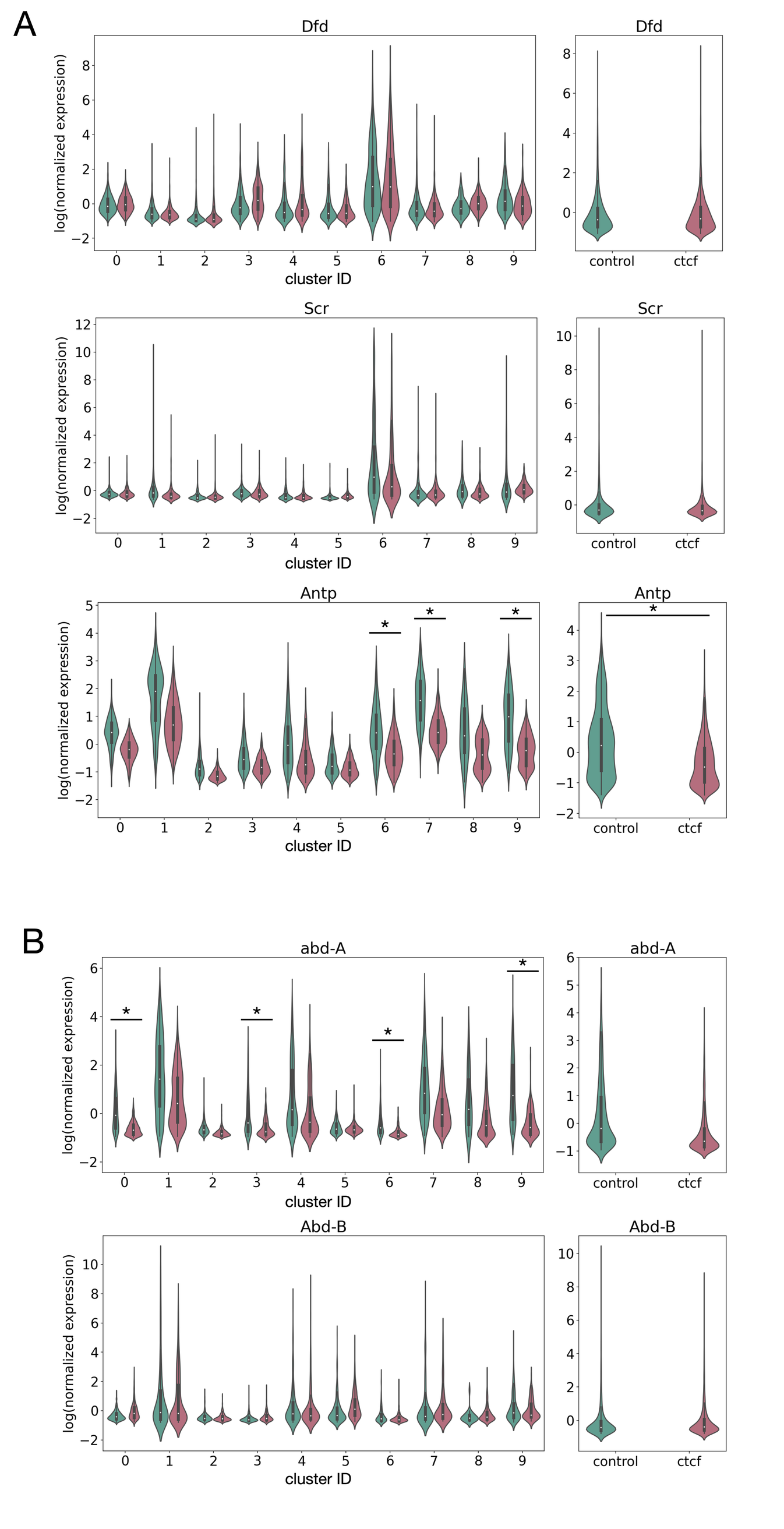

Supplement: S10 Fig — Plots of log(scvi normalized expression) of select (A) Antennapedia complex genes: Dfd (top), Scr (middle), and Antp (bottom) and (B) Bithorax complex genes: abd-A (top) and Abd-B (bottom) in each cluster (left) and in bulk (right) for control (teal) and dCTCFmat-/- nuclei (pink). Asterisks indicate statistically significant differential expression (absolute value of expression > = 1.5 and Bonferroni corrected p-value < 0.05). (TIF) [file pone.0270471.s011.tif]
