# Supplementary material for: Single-nucleus RNA-sequencing in pre-cellularization Drosophila melanogaster embryos
Source: PLoS One. 2022 Jun 24;17(6):e0270471. doi: 10.1371/journal.pone.0270471 (PMC9232161; doi:10.1371/journal.pone.0270471)
Supplement: Attachment — Submitted filename: 20220607_revision_response.docx [file pone.0270471.s013.docx]

*[Note: HTML markup is below. Please do not edit.]*

*Reviewers' comments:*

*Reviewer's Responses to Questions*

***Comments to the Author***

*1. If the authors have adequately addressed your comments raised in a previous round of review and you feel that this manuscript is now acceptable for publication, you may indicate that here to bypass the “Comments to the Author” section, enter your conflict of interest statement in the “Confidential to Editor” section, and submit your "Accept" recommendation.*

*Reviewer #1: (No Response)*

*2. Is the manuscript technically sound, and do the data support the conclusions?*

*The manuscript must describe a technically sound piece of scientific research with data that supports the conclusions. Experiments must have been conducted rigorously, with appropriate controls, replication, and sample sizes. The conclusions must be drawn appropriately based on the data presented.*

*Reviewer #1: Yes*

*3. Has the statistical analysis been performed appropriately and rigorously?*

*Reviewer #1: Yes*

*4. Have the authors made all data underlying the findings in their manuscript fully available?*

*The* [*PLOS Data policy*](http://www.plosone.org/static/policies.action#sharing) *requires authors to make all data underlying the findings described in their manuscript fully available without restriction, with rare exception (please refer to the Data Availability Statement in the manuscript PDF file). The data should be provided as part of the manuscript or its supporting information, or deposited to a public repository. For example, in addition to summary statistics, the data points behind means, medians and variance measures should be available. If there are restrictions on publicly sharing data—e.g. participant privacy or use of data from a third party—those must be specified.*

*Reviewer #1: Yes*

*5. Is the manuscript presented in an intelligible fashion and written in standard English?*

*PLOS ONE does not copyedit accepted manuscripts, so the language in submitted articles must be clear, correct, and unambiguous. Any typographical or grammatical errors should be corrected at revision, so please note any specific errors here.*

*Reviewer #1: Yes*

*6. Review Comments to the Author*

**1. Lines 212-213: Related to my original points 1+2, this sentence could cause confusion if the reader interprets this as meaning that snRNA-seq was performed prior to zygotic genome activation. In this study, snRNA-seq was performed on embryos undergoing zygotic genome activation; therefore, dCTCF[mat-/-] embryos probably transcribe CTCF mRNA (this could be verified in the snRNA-seq data) but do not translate (at least high levels of) CTCF protein yet. This could be explained more clearly.**

We agree that we could better explain the timing of our collection and the assumptions we were working under. We added language to the figure legend for S1 Fig, “The 2h embryos were aged for an additional 2 hours with the majority of the embryos representing nuclear cycle 14, the same time point at which we conducted single-nucleus RNA-sequencing,” in order to clarify that we did not detect CTCF protein at this time point.

As you have alluded to, we are working under the assumption that any zygotically expressed CTCF would not have time to be translated, folded, and function. For this reason, we never intended to genotype the embryos for zygotic dCTCF. In the figures pasted below, we do see low CTCF expression (average 0.81 TPM); however, we only detected expression in 270 control and 467 nuclei (less than 10% of our total nuclei). Although expression appears higher in the dCTCF[mat-/-] embryos and in individual clusters by eye, these differences are not statistically significant. We decided not to include these panels in S1 Fig as the Western blot definitively shows the absence of dCTCF protein in the early embryo.

As far as what we originally had in 212-213, we decided to remove those sentences entirely in order to avoid the confusion. We believe that the Western blot is enough to show that dCTCF protein will have no effect on our results, because we did not detect it, without speculating on the effect of zygotic gene expression.

**2. Lines 56-60: Related to my original point 6, I feel that the authors should tone down their argument that previous scRNA-seq studies may not have primarily measured zygotic expression due to the presence of maternal cytoplasmic RNAs in cells. The present study, Karaiskos et al. 2017, and Ing-Simmons et al. 2021 likely all handled nuclei instead of cells because embryos were dounce-homogenized in all three studies – resulting in nuclei, not intact cells. In my view, the information obtained by snRNA-seq and scRNA-seq in fly embryos is thus comparable (if the authors don’t agree, please explain why). The novelty of the current study is, in my view, rather the demonstration that snRNA-seq is sensitive enough to detect relatively subtle gene misexpression defects in mutant embryos lacking major defects in cell fate decisions.**

This is a great point, we were relying on the assumption that the other works isolated cells rather than nuclei, but realize that this may not necessarily be the case. In order to tone down the language in our manuscript, we removed the statements about zygotic gene expression in lines 56-60 and replaced them with the underlined, “These studies demonstrate the potential for single-cell RNA-sequencing to answer questions relating to pattern and body axis formation in the early *Drosophila* embryo; however, whether single-cell RNA-sequencing is sensitive enough to detect subtle changes in gene expression in mutant embryos lacking major defects remains unclear.”

We believe this change still highlights that our work was conducted earlier in development, as well as detects subtle changes in expression as you mentioned, but removes the unclear language on zygotic gene expression.

**3. Line 317: “Embryonic” should be changed to “larval”, as RNA-seq was performed on central nervous systems of third instar larvae (not embryos) in Kaushal et al. 2021.**

Thank you for catching this, we have fixed this in the manuscript.
